# Social isolation, loneliness, genetic susceptibility, and the hazard of incident osteoporosis

**DOI:** 10.1097/JS9.0000000000003467

**Published:** 2025-09-18

**Authors:** Jian Zhou, Xiaojiang Hu, Shenghua Zhou, Tang Liu, Zhangling Chen

**Affiliations:** a Department of Orthopedics, The Second Xiangya Hospital of Central South University, Changsha, China; b National Clinical Research Center for Mental Disorders, and National Center for Mental Disorders, The Second Xiangya Hospital of Central South University, Changsha, China; c Postdoctoral Mobile Station of Clinical Medicine, The Second Xiangya Hospital of Central South University, Changsha, China; d Department of Cardiovascular Medicine, The Second Xiangya Hospital, Central South University, Changsha, China; e Hunan Key Laboratory of Cardiometabolic Medicine, Changsha, Hunan, China; f FuRong Laboratory, Changsha, China; g Department of Epidemiology, Erasmus MC, University Medical Center Rotterdam, Rotterdam, The Netherlands

**Keywords:** genetic susceptibility, loneliness, osteoporosis, social isolation

## Abstract

**Background:**

To explore the association of social isolation and loneliness with risk of incident osteoporosis; and test the modification effects of genetic susceptibility.

**Methods:**

A total of 452 433 participants without osteoporosis at baseline were included from the UK Biobank cohort. Social isolation and loneliness were assessed at baseline via self-reported questionnaires. Cox regression models were used to observe the associations of social isolation and loneliness with risk of incident osteoporosis, as well as their joint associations with genetic susceptibility.

**Results:**

During a median follow-up of 13.8 years, a total of 13 817 incident osteoporosis cases were recorded. Compared with participants with a social isolation index of 0, those with an index ≥2 had a higher risk of osteoporosis, with a hazard ratio (HR) of 1.18 (95% CI, 1.11-1.25) and a *P*-trend < 0.001. Compared to participants with an index of 0 for loneliness, the HR and 95% CI were 1.25 (95% CI, 1.17, 1.34) for those with a loneliness index of 2 (*P*-trend < 0.001). In addition, we found that the increased risks of osteoporosis related to social isolation and loneliness were strengthened by the genetic susceptibility to osteoporosis.

**Conclusions:**

Our findings suggest that social isolation and loneliness are related to a higher risk of incident osteoporosis, and the associations were strengthened by the genetic susceptibility to osteoporosis.

## Introduction

Osteoporosis is a systemic skeletal disorder characterized by decreased bone mineral density and structural deterioration^[1,2]^. Currently, osteoporosis has become a major public health issue worldwide. The global annual incidence of osteoporosis has reached approximately 41.5 million cases^[3]^, which has imposed substantial medical costs and socioeconomic burdens due to its strong associations with severe chronic pain, reduced mobility, and significant declines in quality of life^[4]^.

**HIGHLIGHTS**

- Social isolation and loneliness were related to an increased risk of incident osteoporosis.
- Indicators of social isolation and loneliness showed consistent associations with the risk of incident osteoporosis.
- Genetic susceptibility strengthened the associations of social isolation and loneliness with the risk of incident osteoporosis.

Efforts to address social isolation and loneliness have been ongoing for decades. The U.S. Surgeon General has emphasized the pressing need to tackle the public health crisis caused by loneliness^[5,6]^. Recently, observational studies have indicated that social isolation and loneliness are associated with an elevated risk of fractures and reduced bone mineral density (BMD), which are closely related to osteoporosis^[7]^. In addition, individuals experiencing social isolation and loneliness are more likely to have lower levels of daily physical activity and more sedentary time, both major risk factors for osteoporosis^[8–10]^. However, to our knowledge, no previous prospective cohort studies have examined the associations of social isolation and loneliness with risk of osteoporosis, and to date, whether and how social isolation and loneliness impact osteoporosis remains unknown.

In this study, we aimed to examine the associations of social isolation and loneliness with the risk of incident osteoporosis using data from the UK Biobank. We also investigated the combined impact of social isolation, loneliness and genetic susceptibility on incident osteoporosis. We hypothesized that social isolation and loneliness were associated with a higher risk of incident osteoporosis, and the associations were strengthened by the genetic susceptibility to osteoporosis.

## Methods

This cohort study has been reported in line with the revised STROCSS 2025 guidelines^[11]^. The UK Biobank study received approval from the National Health and Social Care Information Governance Committee and the Northwest Multi-Centre Research Ethics Committee. All participants provided written informed consent at the time of recruitment^[12,13]^.

### Population for analysis

The UK Biobank is a large-scale prospective population cohort study, with over 500,000 participants aged between 40 and 70 years enrolled between 2006 and 2010^[12]^. From the initial 502 366 individuals registered in the UK Biobank, we excluded 35 707 participants without information on exposure. We further excluded participants with a BMD T-score ≤2.5 (*n* = 5807), those who self-reported osteoporosis at baseline (*n* = 6842), or those diagnosed with osteoporosis at baseline (*n* = 1577). Finally, a total of 452 433 participants were included in the primary analysis (Supplementary Digital content, Figure 1, available at: http://links.lww.com/JS9/F189).

**Figure 1.**
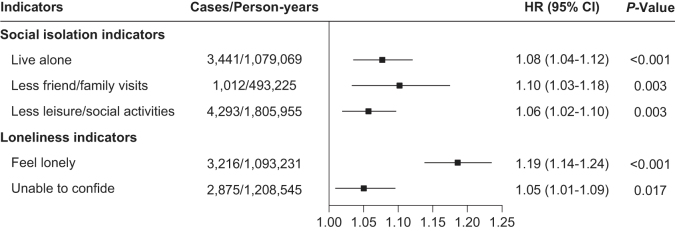
Association of individual components of social isolation and loneliness with risk of incident osteoporosis via multivariable model. Multivariable model were adjusted for age (years), sex (male or female), ethnic background (white, mixed, Asian or Asian British, black or black British, Chinese or other ethnic group), Townsend deprivation index, education years (<15, 15 to <20, or ≥20 years), body mass index (<18.5, 18.5 to <30, or ≥30 kg/m^2^), smoking status (never, previous, or current smoking), alcohol intake frequency (<3 or ≥3 times/week), healthy diet score (<3 or ≥3) and physical activity (<150 min/week or ≥150 min/week), anxiety (no or yes), depression (no or yes), having seen a psychiatrist (no or yes), dementia (no or yes), glucocorticoid use (no or yes), vitamin D supplementation (no or yes), and calcium supplementation (no or yes).

### Assessment of social isolation and loneliness

Social isolation and loneliness were assessed using self-reported questionnaires of UK Biobank^[13,14]^ (Supplementary Digital content, Table 1, available at: http://links.lww.com/JS9/F189). The social isolation index was assessed using three questions. (1) “Including yourself, how many people are living together in your household?”; (2) “How often do you visit friends or family or have them visit you?”; (3) “Which of the following (sports club or gym, pub or social club, religious group, adult education class, other group activity) do you attend once a week or more often?.” The responses including living alone, having friends and family visit less than once a month, and no participating in social activity at least once per week were defined as high-risk factors, which were coded as index = 1, and those responses with low-risk factors were coded as index = 0. The loneliness index was assessed via two questions: (1) “Do you often feel lonely?”; (2) “How often are you able to confide in someone close to you?.” The responses including feeling lonely once every few months/never or almost never being able to confide were defined as high-risk factors, which were coded as index = 1, and those responses with low-risk factors were coded as index = 0. Given the relatively small number of participants in social isolation index = 3 group, participants with index = 3 were combined into index ≥2 group. We calculated the total indexes of social isolation and loneliness by summing the individual indexes of the three and two corresponding indicators, respectively, with a range of 0 to 2 or greater and 0 to 2. Individuals were defined as being social isolation or loneliness if they had a total score of ≥2. Detailed evaluation for social isolation and loneliness is presented in Supplementary Digital content, Table 1, available at: http://links.lww.com/JS9/F189

**Table 1:** Baseline features of participants

Characteristics	Social isolation			Loneliness		
	Social isolation index = 0	Social isolation index = 1	Social isolation index≥2	Loneliness index = 0	Loneliness index = 1	Loneliness index = 2
	(*n* = 239 452)	(*n* = 172 202)	(*n* = 40 779)	(*n* = 306 335)	(*n* = 117 794)	(*n* = 28 304)
Age, years, mean (SD)	56.43 (8.11)	56.26 (8.11)	56.46 (7.80)	56.38 (8.10)	56.51 (8.08)	55.74 (7.93)
Female, *n* (%)	128 704 (53.75)	94 464 (54.86)	20 809 (51.03)	166 930 (54.49)	62 437 (53.01)	14 610 (51.62)
Ethnic background, *n* (%)						
Other	19 453 (8.12)	15 266 (8.87)	4741 (11.63)	24 530 (8.01)	11 901 (10.11)	3029 (10.7
White	219 999 (91.88)	156 936 (91.13)	36 038 (88.37)	281 805 (91.99)	105 893 (89.9)	25 275 (89.3)
Townsend Deprivation Index, mean (SD)	−1.82 (2.784)	−1.097 (3.137)	0.016 (3.493)	−1.605 (2.901)	−1.015 (3.22)	−0.458 (3.427)
Education, years, *n* (%)						
<15	85 342 (35.87)	70 935 (41.53)	18 222 (45.14)	110 482 (36.3)	50 750 (43.49)	13 267 (47.37)
15–19	68 761 (28.91)	45 990 (26.93)	9801 (24.28)	84 990 (27.92)	31 944 (27.37)	7618 (27.21)
≥20	83 789 (35.22)	53 876 (31.54)	12 342 (30.58)	108 883 (35.77)	34 004 (29.14)	7120 (25.42)
Body mass index (kg/m^2^), *n* (%)						
<18.5	895 (0.38)	855 (0.5)	324 (0.8)	1360 (0.45)	540 (0.46)	174 (0.62)
18.5- <30	184 089 (77.17)	125 750 (73.39)	28 299 (69.91)	234 232 (76.76)	84 963 (72.54)	18 943 (67.4)
≥30	53 572 (22.46)	44 745 (26.11)	11 855 (29.29)	69 555 (22.79)	31 629^[26]^	8988 (31.98)
Smoking status, *n* (%)						
Never	135 658 (56.8)	91 866 (53.53)	19 595 (48.27)	170 599 (55.85)	62 668 (53.38)	13 852 (49.14)
Previous	84 258 (35.28)	59 485 (34.66)	13 465 (33.17)	107 569 (35.21)	40 151 (34.2)	9488 (33.66)
Current	18 926 (7.92)	20 269 (11.81)	7531 (18.55)	27 301 (8.94)	14 578 (12.42)	4847 (17.2)
Alcohol intake, times/week, *n* (%)						
<3	122 560 (51.2)	102 326 (59.46)	27 483 (67.47)	163 425 (53.37)	70 698 (60.07)	18 246 (64.54)
≥3	116 809 (48.8)	69 778 (40.54)	13 248 (32.53)	142 812 (46.63)	46 997 (39.93)	10 026 (35.46)
Healthy diet score, *n* (%)						
<3	74 605 (32.01)	56 651 (34.15)	14 339 (37.18)	93 801 (31.5)	40 796 (36.12)	10 998 (41.09)
≥3	158 481 (67.99)	109 230 (65.85)	24 232 (62.82)	204 024 (68.5)	72 152 (63.88)	15 767 (58.91)
Physical activity, min/week, *n* (%)						
<150	57 026 (28.03)	45 731 (34.96)	10 972 (39.3)	77 402 (30.85)	29 293 (32.24)	7034 (34.43)
≥150	146 444 (71.97)	85 069 (65.04)	16 945 (60.7)	173 486 (69.15)	61 578 (67.76)	13 394 (65.57)
Depression, n (%)						
<3	227 818 (95.14)	160 701 (93.32)	36 684 (89.96)	293 754 (95.89)	107 272 (91.07)	24 177 (85.42)
≥3	11 634 (4.86)	11 501 (6.68)	4095 (10.04)	12 581 (4.11)	10 522 (8.93)	4127 (14.58)
Anxiety, *n* (%)						
No	235 853 (98.5)	168 874 (98.07)	39 711 (97.38)	302 122 (98.62)	114 961 (97.59)	27 355 (96.65)
Yes	3599 (1.5)	3328 (1.93)	1068 (2.62)	4213 (1.38)	2833 (2.41)	949 (3.35)
Having seen a psychiatrist, *n* (%)						
No	215 856 (90.37)	150 361 (87.66)	33 429 (82.47)	279 166 (91.37)	99 231 (84.64)	21 249 (75.56)
Yes	22 998 (9.63)	21 159 (12.34)	7107 (17.53)	26 381 (8.63)	18 011 (15.36)	6872 (24.44)
Dementia, n (%)						
No	239 359 (99.96)	172 130 (99.96)	40 756 (99.94)	306 217 (99.96)	117 740 (99.95)	28 288 (99.94)
Yes	93 (0.04)	72 (0.04)	23 (0.06)	118 (0.04)	54 (0.05)	16 (0.06)
Glucocorticoid use, *n* (%)						
No	230 663 (96.33)	165 453 (96.08)	39 085 (95.85)	294 886 (96.26)	113 192 (96.09)	27 123 (95.83)
Yes	8789 (3.67)	6749 (3.92)	1694 (4.15)	11 449 (3.74)	4602 (3.91)	1181 (4.17)
Calcium supplementation, *n* (%)						
No	224 747 (93.99)	161 209 (93.77)	38 025 (93.54)	287 245 (93.88)	110 204 (93.77)	26 532 (94.02)
Yes	14 382 (6.01)	10 715 (6.23)	2628 (6.46)	18 710 (6.12)	7328 (6.23)	1687 (5.98)

### Assessment of incident osteoporosis

Information on incident osteoporosis was defined by analyzing hospital inpatient records, which included data on admissions and diagnoses from the Hospital Episode Statistics for England, Scottish Morbidity Record data for Scotland, and the Patient Episode Database for Wales^[15]^. Participants with osteoporosis were identified based on International Classification of Diseases, 10th Revision (ICD-10) codes (M80–M82). The timing of incident osteoporosis was derived from cumulative medical records of hospital diagnoses, collected until 19 December 2022. The follow-up duration was determined starting from the recruitment date to either the first osteoporosis diagnosis, loss of follow-up, death, or the conclusion of the current monitoring period, whichever occurred first.

### Genetic risk scores

The genotyping procedures and quality control measures for the UK Biobank have been detailed in previous publications^[16]^. In this research, we derived the Genetic Risk Score for osteoporosis (osteoporosis-GRS) from the UK Biobank, which is accessible via its research access platform. The osteoporosis-GRS was calculated using effect sizes from genome-wide association studies (GWAS) summary statistics, multiplied by allele dosage across the genome to obtain an individual-level score. Participants were then stratified into three genetic risk categories (low, medium, and high) based on tertiles of the GRS distribution within our study population, with the lowest tertile representing low genetic risk, the intermediate tertile representing medium genetic risk, and the highest tertile representing high genetic risk.

### Covariates

Age, sex, ethnic background, Townsend deprivation index, education years, BMI, smoking status, alcohol intake frequency, having seen a psychiatrist, vitamin D supplementation and calcium supplementation were collected using questionnaires at baseline. Healthy diet score was calculated according to the consumption of vegetable, fruit, fish, processed meat, and unprocessed red meat (Supplement Digital content, Table 2, available at: http://links.lww.com/JS9/F189) following our previous study^[17]^, with a range of 0–5 score. We classified participants into two groups based on their total moderate physical activity minutes per week, following global recommendations for physical activity and health^[8]^. One minute of vigorous physical activity was considered equivalent to 2 min of moderate physical activity. The two groups were defined as follows: < 150 or ≥150 min/week of physical activity. Depression and anxiety were defined as having a self-reported history or being diagnosed using ICD-10 codes.

**Table 2:** Associations of social isolation and loneliness with risk of incident osteoporosis

	Index = 0	Index = 1	Index≥2	*P*-trend
Social isolation				
Cases, n	6725	5539	1553	
Person-years	3 205 626	2 283 068	528 593	
Model 1	1.00 (reference)	1.16 (1.12–1.20)	1.42 (1.34–1.50)	<0.001
Model 2	1.00 (reference)	1.15 (1.11–1.19)	1.44 (1.37–1.53)	<0.001
Model 3	1.00 (reference)	1.07 (1.03–1.11)	1.22 (1.15–1.29)	<0.001
Model 4	1.00 (reference)	1.05 (1.02–1.09)	1.18 (1.11–1.25)	<0.001
Loneliness				
Cases, n	8813	3917	1087	
Person-years	4 087 390	1 558 027	371 874	
Model 1	1.00 (reference)	1.17 (1.13–1.22)	1.36 (1.28–1.45)	<0.001
Model 2	1.00 (reference)	1.16 (1.12–1.21)	1.44 (1.36–1.54)	<0.001
Model 3	1.00 (reference)	1.11 (1.07–1.15)	1.33 (1.25–1.42)	<0.001
Model 4	1.00 (reference)	1.07 (1.03–1.12)	1.25 (1.17–1.34)	<0.001

Model 1: Unadjusted model.

Model 2: Adjusted for age (years) and sex (male or female).

Model 3: Model 2 + ethnic background (white, mixed, Asian or Asian British, black or black British, Chinese or other ethnic group), Townsend deprivation index, education years (<15, 15 to <20 or ≥20 years), body mass index (<18.5, 18.5 to <30, or ≥30 kg/m^2^), smoking status (never, previous, or current smoking), alcohol intake frequency (<3 or ≥3 times/week), healthy diet score (<3 or ≥3), and physical activity (<150 min/week or ≥150 min/week).

Model 4: Model 3 + anxiety (no or yes), depression (no or yes), having seen a psychiatrist (no or yes), dementia (no or yes), glucocorticoid use (no or yes), vitamin D supplementation (no or yes), calcium supplementation (no or yes).

### Statistical analysis

Continuous variables were presented as mean ± standard deviation, while categorical variables were described as counts (percentages). The associations of social isolation and loneliness with osteoporosis were assessed using Cox proportional hazard regression models. The proportionality of hazards was authenticated via Schoenfeld residuals and Kaplan–Meier methods^[18]^, with all analyses adhering to predefined conditions. Model 1 was an unadjusted model. Model 2 was adjusted for age (years) and sex (male or female). Model 3 was further adjusted for ethnic background (White, Mixed, Asian or Asian British, Black or Black British, Chinese or other ethnic group), Townsend deprivation index, education years (<15, 15 to <20, or ≥20 years), body mass index (<18.5, 18.5 to <30, or ≥30 kg/m^2^), smoking status (never, previous, or current), alcohol intake frequency (<3 or ≥3 times/week), healthy diet score (<3 or ≥3), and physical activity (<150 min/week or ≥150 min/week). Model 4 was additionally adjusted for anxiety (no or yes), depression (no or yes), history of psychiatric consultation (no or yes), dementia (no or yes), glucocorticoid use (no or yes), vitamin D supplementation (no or yes), and calcium supplementation (no or yes). Also, we examined the associations of the specific components of social isolation and loneliness, including live alone, less friends/family visits, less leisure/social activities, feel lonely, and unable to confide with risk of incident osteoporosis. Furthermore, we conducted joint analyses to evaluate the combined impact of social isolation and loneliness with osteoporosis-GRS on the incidence of osteoporosis. We also examined a joint analysis of social isolation and loneliness with incident osteoporosis risk.

Moreover, we conducted a series of subgroup analyses stratified by age (<60 or ≥60 years), sex (female or male), ethnic background (white or other), Townsend Deprivation Index (<median or ≥median), education years (<10 or ≥10 years), BMI (<25, 25 to <30, or ≥30 kg/m^2^), smoking status (never, previous, or current) alcohol consumption frequency (<3 or ≥3 times/week), healthy diet score (<3 or ≥3), physical activity level (<150 or ≥150 min/week), and the presence of anxiety (no or yes), depression (no or yes), having seen a psychiatrist (no or yes), vitamin D supplementation (no or yes), and calcium supplementation (no or yes).

In addition, sensitivity analyses were performed to test the robustness of our results. First, we repeated the analyses by excluding participants who developed osteoporosis within the first 2 years of follow-up. Second, social isolation and loneliness were mutually adjusted for each other. Third, participants with missing covariate data were removed. Finally, we used multiple imputation methods to address missing covariate data. The numbers and percentages of participants with missing covariates were provided in Supplementary Digital content, Table 3, available at: http://links.lww.com/JS9/F189. All statistical analyses were performed using SAS version 9.4 (SAS Institute, Cary, NC, USA), and significance was defined as a two-sided *P* < 0.05.

## Results

### Baseline characteristic

The baseline characteristics of the included participants are presented in Table 1. Participants experiencing higher levels of social isolation or loneliness were more likely to be non-White, have a higher Townsend Deprivation Index, fewer years of education, a higher BMI, be current smokers, consume less alcohol, have lower diet quality, engage in less physical activity, and be more likely to report depression, anxiety, psychiatric consultations, and vitamin D or calcium supplementation.

### Associations of social isolation and loneliness with the risk of incident osteoporosis

During a median follow-up time of 13.8 (IQR: 12.9, 14.5) years, we recorded a total of 13 817 incident osteoporosis cases among the 452 433 participants. Multivariable adjusted hazard ratios (HRs) for osteoporosis were 1.05 (95% CI, 1.02–1.09) and 1.18 (95% CI, 1.11–1.25) for participants with a social isolation index of 1 and ≥2 (*P-*trend *<* 0.001), respectively, compared with those with a social index of 0. Compared with participants with an index of 0 for loneliness, the HRs were 1.07 (95% CI, 1.03–1.12) and 1.25 (95% CI, 1.17–1.34) for participants with a loneliness index of 1 and 2 (*P-trend* < 0.001), respectively (Table 2). Supplementary Digital content, Figure 2, available at: http://links.lww.com/JS9/F189 presents the cumulative hazard curves for the risk of osteoporosis in participants stratified by levels of social isolation and loneliness. The respective cumulative curves for participants with the highest social isolation or loneliness index indicated the highest osteoporosis risk throughout the follow-up period.

**Figure 2.**
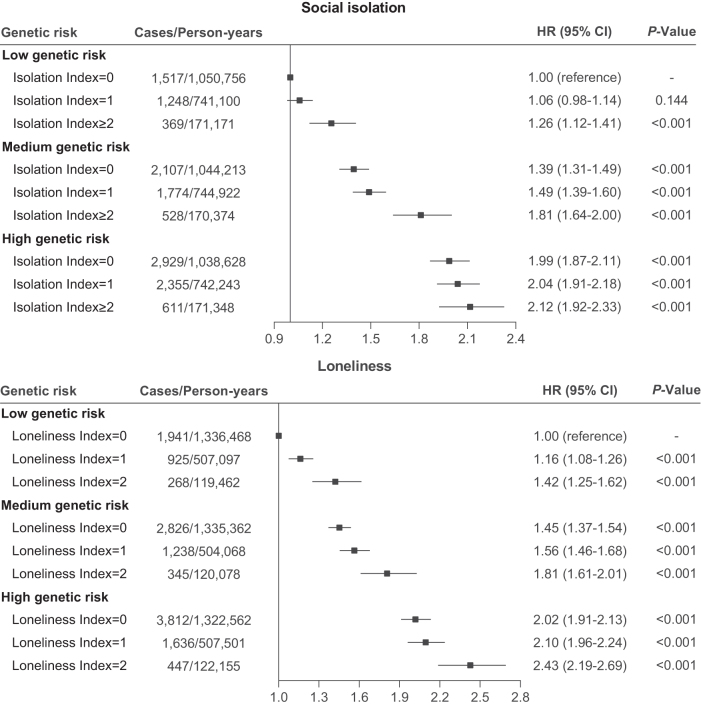
Joint association of social isolation and loneliness with osteoporosis-GRS in relation to risk of incident osteoporosis via multivariable model. Multivariable model were adjusted for age (years), sex (male or female), ethnic background (white, mixed, Asian or Asian British, black or black British, Chinese, or other ethnic group), Townsend deprivation index, education years (<15, 15 to <20, or ≥20 years), body mass index (<18.5, 18.5 to < 30, or ≥30 kg/m^2^), smoking status (never, previous, or current smoking), alcohol intake frequency (<3 or ≥3 times/week), healthy diet score (<3 or ≥3) and physical activity (<150 min/week or ≥150 min/week), anxiety (no or yes), depression (no or yes), having seen a psychiatrist (no or yes), dementia (no or yes), glucocorticoid use (no or yes), vitamin D supplementation (no or yes), and calcium supplementation (no or yes).

Furthermore, the three components of social isolation: live alone, less friends/family visits, and less leisure/social activities were associated with a 6–10% higher risk of osteoporosis. Also, the two components of loneliness indicators: feel lonely and unable to confide were associated with a 19% and 5% higher risk of osteoporosis, respectively (Fig. 1).

### Joint associations of social isolation, loneliness, and osteoporosis-GRS with risk of incident osteoporosis

Joint association analysis showed that that individuals with both high osteoporosis-GRS and the highest social isolation or loneliness index had the most elevated osteoporosis risk. Participants with high osteoporosis-GRS and the highest social isolation or loneliness index faced a 112% (95% CI: 92–133%) and 143% (95% CI: 119–169%) higher risk of incident osteoporosis for social isolation and loneliness, respectively, compared with those with low osteoporosis-GRS and the lowest social isolation or loneliness index (Fig. 2).

### Joint associations of social isolation, loneliness, with risk of incident osteoporosis

**Figure 3.**
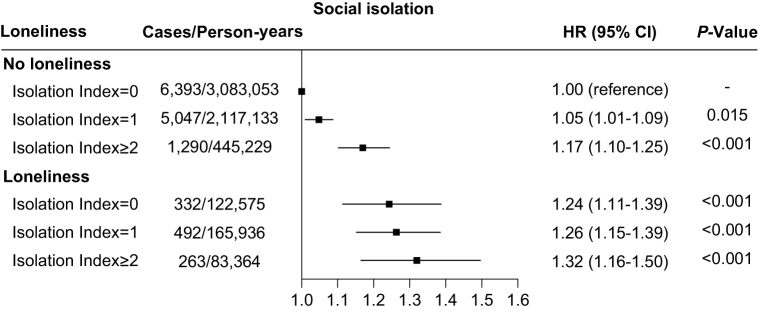
Joint associations of social isolation and loneliness with risk of incident osteoporosis via multivariable model. Multivariable model were adjusted for age (years), sex (male or female), ethnic background (white, mixed, Asian or Asian British, black or black British, Chinese, or other ethnic group), Townsend deprivation index, education years (<15, 15 to <20, or ≥20 years), body mass index (<18.5, 18.5 to <30, or ≥30 kg/m^2^), smoking status (never, previous, or current smoking), alcohol intake frequency (<3 or ≥3 times/week), healthy diet score (<3 or ≥3) and physical activity (<150 min/week or ≥150 min/week), anxiety (no or yes), depression (no or yes), having seen a psychiatrist (no or yes), dementia (no or yes), glucocorticoid use (no or yes), vitamin D supplementation (no or yes), and calcium supplementation (no or yes).

Figure 3 illustrates the combined impact of social isolation and loneliness on the development of osteoporosis. The baseline group consisted of individuals experiencing the lowest levels of isolation and no loneliness. A rising pattern in HRs was noted as the degree of social isolation increased, regardless of loneliness status. Individuals who were both lonely and had a social isolation index of 2 or higher were found to have a 32% increased risk (95% CI: 16–50%) of developing osteoporosis compared to those with lowest levels of isolation and no loneliness (Fig. 3).

### Stratified analyses results

The association of social isolation with risk of incident osteoporosis was consistent across all predefined risk factors except for sex (*P*-interaction <0.001) and smoking status (*P*-interaction = 0.004). The association of isolation was stronger among the male participants or current smokers (Supplementary Digital content, Table 4, available at: http://links.lww.com/JS9/F189). The association of loneliness was also consistent across all risk factors except for age, sex, ethnic background, alcohol intake, and use of vitamin D supplements. The association between loneliness and the risk of incident osteoporosis was more pronounced in participants aged <60 years, male participants, White individuals, those with an alcohol intake frequency of ≥3 times/week, or those not taking vitamin D supplements (*P*-interaction <0.05) (Supplementary Digital content, Table 5, available at: http://links.lww.com/JS9/F189).

### Sensitivity analyses results

The results remained similar when excluding participants who developed osteoporosis within the first 2 years of follow-up; when mutual adjustment for social isolation and loneliness; when excluding participants with missing covariate data; and when imputing missing values for all covariates using multiple imputation (Supplementary Digital content, Tables 6–9, available at: http://links.lww.com/JS9/F189).

## Discussion

In this large population-based prospective cohort study of UK Biobank participants, we observed that both social isolation and loneliness significantly increased the risk of osteoporosis. The more specific components of social isolation and loneliness, including live alone, less friends/family visits, less leisure/social activities, feel lonely, and unable to confide were all associated with a higher risk of osteoporosis. We observed that the associations of social isolation and loneliness with osteoporosis were strengthened by genetic susceptibility to osteoporosis. In addition, these associations were independent of established and potential osteoporosis risk factors and robust in various sensitivity analyses.

To date, only a few population-based cohort studies have reported on the potential association of social isolation and loneliness with bone health^[8-10]^. A cohort study of 11 344 participants from the Canadian Longitudinal Study on Aging reported that social isolation was significantly associated with an increased risk of fractures in older adults, while no association with three-year changes in bone mineral density (BMD) was observed^[9]^. However, this study only assessed the relationship between social isolation and BMD over a short follow-up period of 3 years, which may not be sufficient to capture long-term changes in bone health. Another small cohort study of 146 participants from the UK found that social isolation was associated with a history of fracture in older adults^[9]^. While previous studies have linked social isolation to fracture risk and bone density loss, our study is the first (to our knowledge) to demonstrate an association with clinically diagnosed incident osteoporosis. Leveraging the large sample size, longer duration of follow-up, and rich data on exposures, outcomes, and covariates, we made some novel observations. We observed harmful associations of social isolation and loneliness with risk of osteoporosis, which were attributable to each component of social isolation and loneliness, including live alone, less friends/family visits, less leisure/social activities, feel lonely, and unable to confide. To date, this is the first large-scale cohort study to comprehensively examine the associations of social isolation, loneliness, and their specific components with risk of osteoporosis, which added important evidence into this field and had important clinical and public health implications. Furthermore, this is also the first to investigate the combined impacts of genetic risk, social isolation, and loneliness on osteoporosis risk, and we observed the impact of social isolation and loneliness on osteoporosis risk was more pronounced in individuals with higher genetic risk. A possible explanation is that individuals with a high genetic predisposition are already at a disadvantage in terms of bone metabolism^[19,20]^. When these individuals experience social isolation or loneliness, psychosocial stress may accelerate bone loss or influence the expression of bone metabolism-related genes, thereby exacerbating the progression of osteoporosis^[21]^.

We also observed that the association between social isolation and osteoporosis was stronger among men and current smokers. One possible explanation is that men may respond to social isolation with behaviors more detrimental to bone health, such as increased alcohol or tobacco use, reduced motivation for physical activity, or less attention to dietary quality, all of which can negatively impact bone density^[22,23]^. In addition, smoking is known to be a significant risk factor for bone loss, and individuals who are socially isolated may have higher smoking rates or face challenges in quitting, further compounding their osteoporosis risk^[24]^. The association between loneliness and osteoporosis was more pronounced in younger participants (<60 years), men, White individuals, frequent alcohol consumers, and those not taking vitamin D supplements. Younger individuals experiencing loneliness may adopt harmful behaviors, such as increased alcohol consumption, that negatively impact bone health^[25]^. Vitamin D supplementation may also obscure the promoting effect of loneliness on osteoporosis^[26]^.

The pathways underlying the associations of social isolation and loneliness with increased risk of osteoporosis remain unclear. Nonetheless, there are several plausible pathways. First, social isolation and loneliness are linked to poorer health behaviors, including reduced physical activity, unhealthy diets, and smoking, all of which are established risk factors for osteoporosis^[27–29]^. Indeed, in our analysis, we observed that the associations were stronger among individuals with smoking. Furthermore, social isolation and loneliness are associated with chronic stress, which triggers heightened inflammatory responses and endocrine dysregulation^[30,31]^. For instance, loneliness can trigger chronic stress, leading to elevated cortisol levels, which negatively affect bone metabolism and lead to bone loss^[32,33]^. Prolonged states of chronic inflammation may also accelerate bone loss by increasing osteoclast activity and reducing osteoblast formation^[34]^. Moreover, animal studies have indicated that social isolation may increase risk of osteoporosis via declines in both trabecular and cortical bone parameters^[8]^. In addition, a lack of social support may prevent individuals from receiving timely medical interventions or preventive measures, further increasing the risk of osteoporosis^[35]^. Taken together, these findings underscore the possible multifaceted pathways through which social isolation and loneliness may negatively affect bone health.

Our study had several strengths, including the prospective design, the large sample size, and the comprehensive and extensive details on covariates and analyses. Furthermore, our findings may have implications for surgical practice, particularly in orthopedics. Bone mineral density is closely associated with the outcomes of orthopedic procedures such as fracture fixation, joint replacement, and spinal surgery. Early identification of individuals at high risk for osteoporosis – especially those who are socially isolated or lonely – can help inform preoperative planning, improve perioperative risk stratification, and support timely preventive interventions. By highlighting modifiable psychosocial risk factors, this study may help reduce the incidence of osteoporotic fractures and improve surgical outcomes. Several limitations warrant mentioning. First, data on the duration of loneliness or the stability of social isolation were not available from the UK Biobank cohort. Second, the data primarily came from the UK Biobank, with the sample consisting mainly of middle-aged and older white individuals, which may limit the generalizability of the findings to other ethnic groups or younger populations. Further investigations should be conducted to examine the associations among different ethnicities and age groups^[36]^. Furthermore, social isolation and loneliness were assessed at baseline only. While repeated measurements would provide more detailed temporal information, our use of baseline data is consistent with prior large-scale epidemiological studies using UK Biobank and similar cohorts^[37,38]^. Notably, these psychosocial factors tend to be relatively stable over time, particularly in older adults^[39]^. Therefore, although some misclassification due to unmeasured changes over time is possible, it is likely non-differential and would bias results toward the null. Finally, this current study was based on an observational design, and the causality cannot be established. Randomized controlled trials may be conducted to confirm causal relationships.

## Conclusion

Our findings indicate that social isolation and loneliness are associated with a higher risk of incident osteoporosis, and the association is strengthened by the genetic susceptibility to osteoporosis. These results highlight the importance of improving social isolation and loneliness in the prevention of osteoporosis.

## Data Availability

The data can be obtained from the corresponding author on request.

## Patient and public involvement

Participants were not involved in the design, development of outcome measures, or other aspects of the conduct of the study.

## Use of Artificial Intelligence (AI)

During the preparation of this manuscript, artificial intelligence (AI) tools (ChatGPT-4o, GPT-4.5, developed by OpenAI) were used solely for language editing and translation purposes. No AI tools were involved in study design, data analysis, result interpretation, or figure generation. All outputs from the AI system were carefully reviewed and verified by the authors, who take full responsibility for the integrity and accuracy of the final content. No identifiable data were shared with the AI system, and all usage complied with relevant privacy regulations.
